# Better Living with Non-memory-led Dementia: study protocol for a randomised controlled trial of a web-based caregiver educational programme (BELIDE trial)

**DOI:** 10.1136/bmjopen-2025-102518

**Published:** 2025-09-05

**Authors:** Aida Suarez-Gonzalez, Emilie Brotherhood, Amber John, Oliver Hayes, Sam Rossi-Harries, Nikki Zimmermann, Valerie Mansfield, Andrew Brand, Zoe Hoare, Deborah Fitzsimmons, Katherine Cullen, Sebastian Crutch, Joshua Stott

**Affiliations:** 1 Dementia Research Centre, UCL Queen Square Institute of Neurology, UCL, London, UK; 2 ADAPTLab, Clinical Educational and Health Psychology, Psychology and Language Sciences, University College London, London, UK; 3 North Wales Organisation for Randomised Trials in Health & Social Care, Bangor University, Bangor, UK; 4 Swansea Centre for Health Economics, Swansea University, Swansea, Wales, UK

**Keywords:** Caregivers, Health Education, Dementia

## Abstract

**Introduction:**

Carers of people with non-memory-led dementias such as posterior cortical atrophy (PCA), primary progressive aphasia (PPA) and behavioural variant frontotemporal dementia (bvFTD) face unique challenges. Yet, little evidence-based support and guidance are available for this population. To address this gap in services, we have developed a novel, web-based educational programme: the Better Living with Non-memory-led Dementia programme (BELIDE). BELIDE was co-designed with people with lived experience of non-memory-led dementia and a previous pilot study confirmed its feasibility as an online intervention. This protocol outlines the randomised controlled trial (RCT) to evaluate the clinical and cost-effectiveness of BELIDE.

**Methods and analysis:**

This is a parallel-group, single-blind, RCT of 238 unpaid caregivers of people diagnosed with PCA, PPA or bvFTD recruited internationally among members of the UK-based organisation Rare Dementia Support. The intervention (BELIDE programme) consists of six structured online educational modules tailored to each phenotype, a virtual onboarding session, real-life practice tasks and up to two follow-up facilitation sessions. The group receiving the intervention will be given access to the programme, while the control group will receive treatment as usual and be placed on a wait-list to receive access to the programme once they complete their participation in the trial. The allocation ratio will be 1:1 stratified by dementia diagnosis and gender. The primary outcome is reduction in caregiver depressive symptoms. Secondary outcomes include stress, anxiety, self-efficacy, quality of life and caregiver-patient relationship quality. Data will be collected online via Qualtrics surveys at baseline, 8 weeks and 6 months post-randomisation. A mixed-method process evaluation with a subgroup of intervention participants will explore barriers and facilitators for engagement. A health economics evaluation will also be conducted to assess cost-effectiveness. If effective, this programme could improve access to caregiver support for non-memory-led dementias by providing scalable, tailored education.

**Ethics and dissemination:**

Ethical approval has been granted by University College London Research Ethics Committee (8545/007). The results will be disseminated via peer-reviewed publications, conferences, stakeholder events and open-access resources.

**Trial registration:**

This trial has been registered prospectively on the Clinical Trials registry, first posted on 5 February 2024 under registration number NCT06241287.

**STRENGTHS AND LIMITATIONS OF THIS STUDY**

- This study uses a randomised controlled trial design with an active waitlist control, stratified randomisation and a large sample of participants with low prevalent dementias, together representing a methodological strength.
- The intervention is delivered fully online, enabling broad geographical reach and consistent delivery and requiring minimum staff time.
- A mixed-methods process evaluation is integrated into the design to explore engagement, implementation and mechanisms of change.
- Web-based delivery increases accessibility but may limit participation for caregivers with low digital literacy.
- Participants are recruited from a specialised support organisation, which may limit representativeness of the wider caregiver population and generalisation of results.

## Introduction

 Non-memory-led dementias initially present with symptoms different from the memory deficits associated with more common phenotypes, such as Alzheimer’s disease. For instance, posterior cortical atrophy (PCA) primarily affects cortical visual abilities,^1^ primary progressive aphasia (PPA) impairs language^2^ and the behavioural variant frontotemporal dementia (bvFTD) leads to behavioural changes.^3^ People with these conditions are more impaired in daily tasks than those with typical dementia.^4^,^8^

These types of dementia often affect those under 65, who are still employed^9^ and managing caregiving and financial responsibilities. Their family members, who usually take on caregiving roles, face disruptions in their own employment and occupational goals.^10^ The lower prevalence and wider geographical spread of these phenotypes, along with their atypical symptoms, make it difficult for caregivers to find reliable, high-quality information and educational resources.^11 12^ Families frequently highlight the need for phenotype-specific support, revealing gaps in education, training and post-diagnostic services within the existing dementia care pathway.^13^,^17^

### Web-based educational resources

Family caregivers’ education and training constitute key tools for tertiary prevention. Appropriate and fit for purpose training equips caregivers with the skills to support the person with dementia and promote their own well-being, reducing the likelihood of disease-related complications. Lower competency in caregiving is linked to reduced quality of life in the person with dementia^18^ and to a higher risk of institutionalisation.^19^ On the other hand, caregivers with a low sense of competency experience more hopelessness and low mood.^20^ Furthermore, there is evidence that coping strategies and cognitive appraisal styles^21^,^25^ can act as mediators between perceived stress and caregivers’ health, and these factors can be modified through training.

Interest in online delivery of educational interventions is growing due to its potential for accessibility, flexibility and sustainability.^26^,^29^ A meta-analysis of online caregiver education tools on health outcomes found a small but significant effect on reducing caregiver depression and a medium effect on reducing caregiver distress, with no effect found on caregiver burden or self-efficacy.^30^ Similarly, a recent Cochrane review found no significant effect of online support and training on the quality of life or health outcomes in informal caregivers of people with dementia.^31^

There are two main online interventions developed to provide education and training to caregivers of people with young-onset and low-prevalent dementias specifically.^32^,^36^ Partner in Balance is a blended (human-digital) educational intervention for carers of people with young-onset dementia, including FTD, adapted from a programme originally designed for early-stage dementia^33^, and RHAPSODY (Research to Assess Policies and Strategies for Dementia in the Young) is a support programme for caregivers of people with young-onset dementia showing good user acceptability, usability and user satisfaction.^26^ However, there is currently not fully powered randomised control trial (RCT) evidence for clinical or cost-effectiveness of these programmes nor is there complete coverage of the non-memory-led dementia spectrum (eg, for PCA). Moreover, Partner in Balance requires ~3 hours of facilitator contact per caregiver and prior facilitator training,^34^ while RHAPSODY is fully self-guided.^35^ Human support may be needed to ensure engagement, as suggested by lower uptake in RHAPSODY and our own feasibility work,^37^ but its costs can limit sustainability. Finding the right balance in the amount of facilitator involvement seems key to effective implementation and roll-out of these programmes.

### Better Living with Non-memory-led Dementia educational programme for caregivers (BELIDE)

Building on previous research, our team developed a novel, blended, web-based caregiver educational programme for families of those with PCA, PPA and bvFTD, the BELIDE programme.^37^ BELIDE is a six-module course co-developed with people with lived experience. It integrates human contact, phenotype-specific information and strategies for symptom management, recommendations for well-being and practical exercises, elements known to enhance adherence in online interventions.^38^ A recent scoping review found that almost half of studies on web-based interventions for informal caregivers of people with dementia were not informed by behaviour change theories.^39^ We sought to do this in BELIDE, which was developed in line with the Medical Research Council (MRC) guidance for development of complex interventions.^40^ BELIDE is informed by a logic model^37^ based on theories of self-efficacy,^23^ behaviour change,^41^ coping theory^24^ and social learning^42^ and modelled according to the COM-B (Capability, Opportunity, Motivation, and Behaviour) model of behaviour change.^41^ The programme is self-administered, minimally supported by a facilitator (a total of 2 hours over two video calls and email interaction) to favour implementation and sustainability. A previous pilot study demonstrated successful recruitment, high completion rates of outcome measures and good acceptability of the BELIDE programme, supporting its use in a fully powered trial.^37^ This manuscript presents the study protocol for a RCT evaluating the effectiveness of BELIDE in improving health outcomes in caregivers of people with PCA, PPA and bvFTD.

### Objectives and research questions

The aims of this trial are (1) to assess the effectiveness and cost-effectiveness of the BELIDE programme in improving psychological outcomes in caregivers of people with non-memory-led dementias and (2) to conduct a mixed-methods process analysis to explore mechanisms of change, barriers and facilitators to access and implementation, as well as perceived benefits and costs of the intervention.

The research questions are the following:

1. Are carer depressive symptoms (primary outcome) significantly reduced in participants allocated to receive BELIDE compared with participants allocated to the control wait-list group receiving treatment as usual (TAU)?
2. Are symptoms of stress and anxiety (secondary outcomes) significantly reduced and caregiver self-efficacy, capability, well-being, quality of carer-patient relationship and quality of life (secondary outcomes) significantly increased in participants allocated to receive BELIDE compared with the control group?
3. How is the BELIDE programme perceived by research participants and what do they perceive as factors and mechanisms influencing engagement, user satisfaction and change?
4. What are the perceived barriers and facilitators for future implementation?
5. What is the cost-effectiveness of BELIDE compared with TAU?

## Methods and analysis

This trial has been registered prospectively in ClinicalTrials.gov (NCT06241287) and prepared according to the Standard Protocol Items: Recommendations for Interventional Trials (SPIRIT)^43^ and the Template for Intervention Description and Replication (TIDieR)^44^ (see online supplemental file 1 for SPIRIT and online supplemental file 2 for TIDieR) and will be reported according to the Consolidated Standards of Reporting Trials.^45 45^ The study commenced in January 2024 and is expected to conclude in September 2027, with primary outcome data collection ending in March 2026.

### Study design

**Figure 1:**
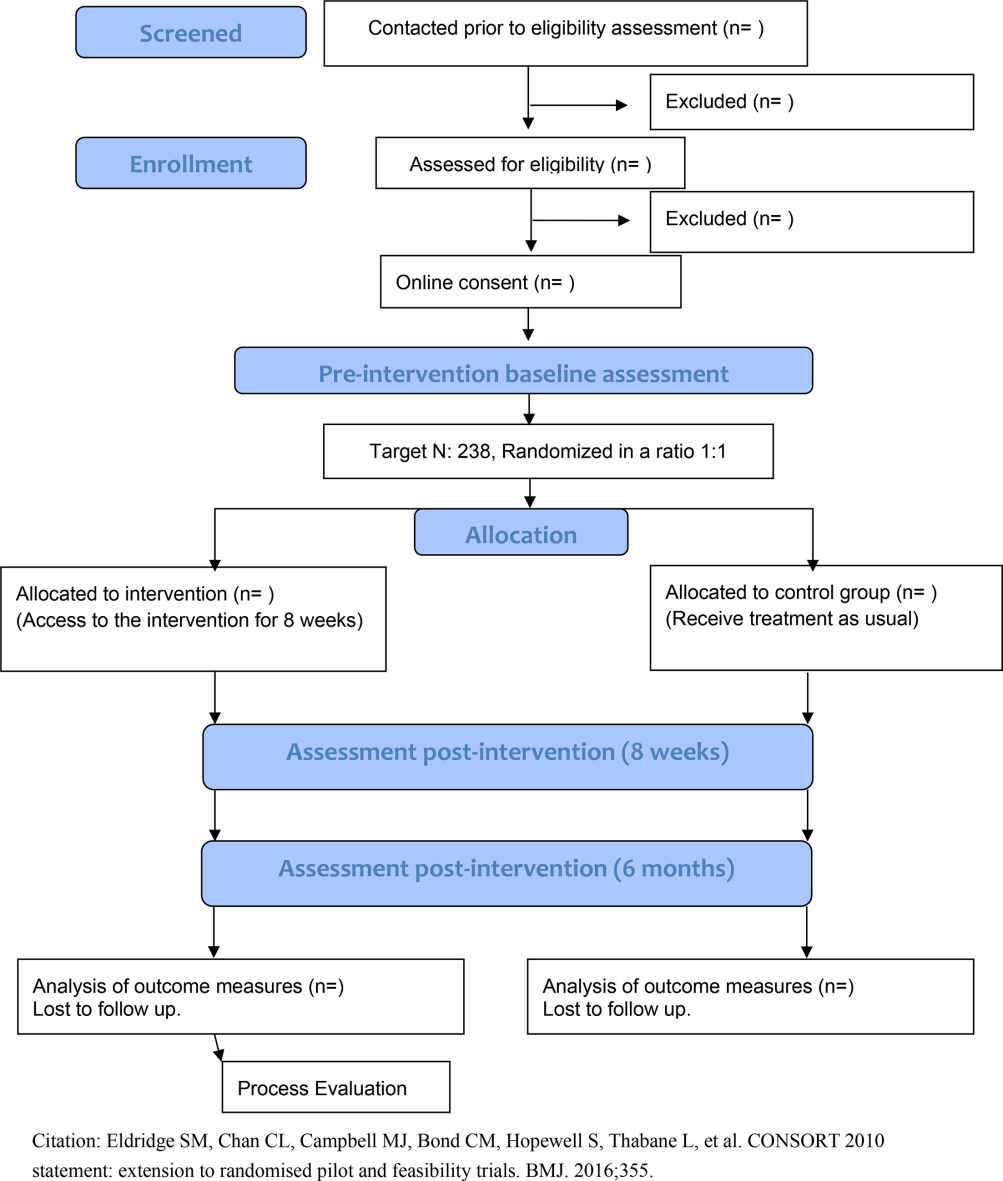
Participant flow chart for Better Living with Non-memory-led Dementia trial.

This is a randomised wait-list control trial assessing the effectiveness of a novel, web-based caregiver educational programme for individuals with non-memory-led dementias, namely, PPA, PCA and bvFTD. Participants will be assigned either to the intervention group, receiving the BELIDE programme over 8 weeks, or the wait-list comparison group, receiving TAU with access to the intervention after the final follow-up measure. The intervention comprises six learning modules delivered via a web-based platform, including virtual onboarding with a facilitator, real-life tasks to apply learnt skills, printable material and virtual check-in sessions with a facilitator. The intervention’s adaptation, design modifications and selection of primary outcome measures were informed by feasibility work.^37^ The study will comprise three workstreams (WS):

- WS1 will evaluate the effectiveness of the BELIDE programme in improving psychological outcomes for caregivers, focusing on reducing depressive symptoms as the primary outcome, with secondary outcomes including reductions in anxiety, stress and improvements in caregiver self-efficacy, relationship quality, well-being and health-related quality of life.
- WS2 is a mixed methods process evaluation to examine perceived costs and benefits of, and mechanisms of change in the intervention as well as participant engagement with the programme, identifying barriers and facilitators for engagement, access and implementation.
- WS3 is a health economic evaluation to assess the cost-effectiveness of the intervention. The study participant flow chart is shown in figure 1.

### Participants

Participants will be recruited internationally among members of the UK-based third sector organisation Rare Dementia Support (RDS) (https://www.raredementiasupport.org/). The trial will recruit unpaid caregivers of individuals diagnosed with non-memory-led dementias, including PPA, PCA and bvFTD. The Direct Support team at RDS will be involved in informing members about the opportunity to take part in this research and its eligibility criteria. The delivery of the educational programme (intervention) will be centralised at University College London (UCL).

Inclusion and exclusion criteria are set out below:

#### Inclusion criteria

1. Adults (18+ years) who self-identify as unpaid carers (with no lower limit on number of hours caring) of individuals with PPA, PCA or bvFTD who are not residing in a full-time care facility.
2. The care recipient must have a confirmed diagnosis of dementia, as reported by the carer (reflecting real-world future implementation).
3. Carers must be able to give informed consent.
4. Carers must have a good understanding of written English.
5. Carers must have access to the internet.

#### Exclusion criteria

1. Carers of individuals living in a full-time care facility.
2. Carers of individuals with severe dementia that significantly impacts activities of daily living (because the intervention is aimed at earlier stage caring).
3. Carers of individuals with any form of dementia other than PPA, PCA or bvFTD.

### Workstream 1 (WS1): effectiveness of the BELIDE educational programme

#### Interventions

##### Experimental group: BELIDE programme

Participants randomised to the intervention group will receive an 8-week structured, web-based educational programme, co-produced with people with lived experience,^37^ designed to provide knowledge, skills and coping strategies to caregivers of people with PCA, PPA and bvFTD. The programme includes six educational modules providing psychoeducation, positive support strategies and self-care techniques and includes real-life ‘put your knowledge into practice’ tasks to reinforce engagement and skill development. The programme is described in full as per the TIDIER checklist (see online supplemental file 2). Briefly, the modules cover the following themes, tailored to each phenotype:

- Introduction to BELIDE and setting expectations
- Understanding the disease
- Providing positive support to the person with dementia
- Caregiver and family mental health and well-being
- Accessing additional sources of support
- The value of support groups

**Figure 2:**
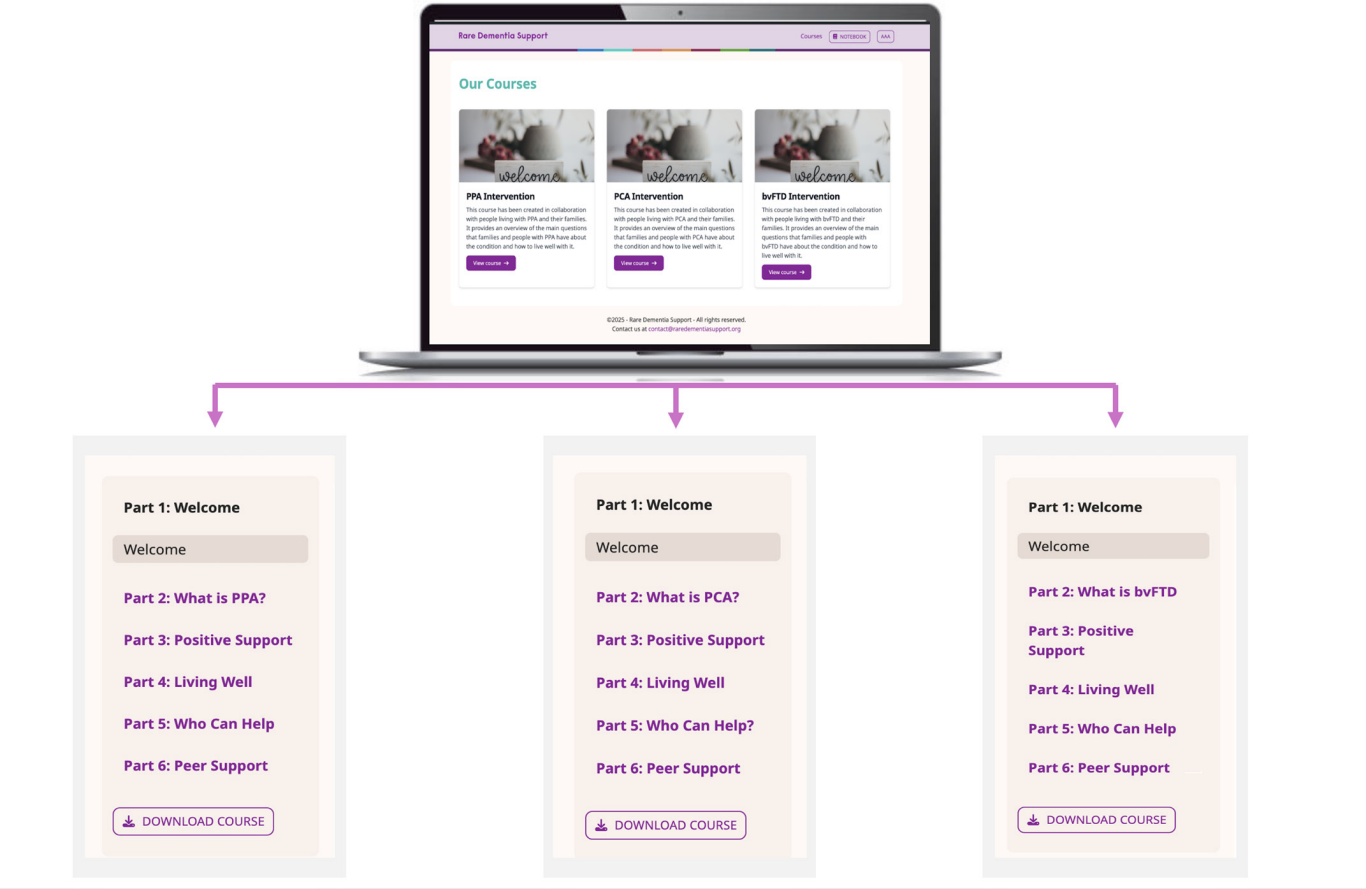
Overview of the BELIDE educational programme. bvFTD, behavioural variant frontotemporal dementia; PCA, posterior cortical atrophy; PPA, primary progressive aphasia.

The programme is delivered entirely online through a web-based platform and participants receive a facilitator-led onboarding session at the start and up to two online check-in sessions (one by Zoom, one by email). The programme is self-paced over 8 weeks. Participants can access all course materials in both the web and printable format. See figure 2 for an overview of BELIDE.

The feasibility study^37^ tested a fully self-guided version of BELIDE, which showed good feasibility of study outcome measures but low adherence and engagement with BELIDE resources. The participants in the feasibility trial suggested ways to improve this, which along with a literature review on online interventions informed our amended approach. To enhance engagement and adherence in the trial, participants must complete an onboarding session with personalised support and troubleshooting before accessing the platform. Additionally, up to two virtual check-ins in the following weeks reinforce accountability and motivation.

##### Control group: wait-list treatment as usual (TAU)

Participants allocated to the control group will receive TAU, which includes explicit signposting to publicly available resources on the RDS website (https://www.raredementiasupport.org/), in addition to any existing support they may already be accessing (eg, counselling, attendance at support groups). To minimise intervention exposure in the control group, participants are only directed to the RDS website. BELIDE content is unique and not publicly available. External resources accessed by the control group are recorded to enable sensitivity analyses.

The control group will have access to the BELIDE web resources (but self-guided, without facilitator input) after their last follow-up assessment (approximately 6 months post randomisation).

### Outcomes

All outcome measures will be collected through UCL’s Qualtrics platform. The primary outcome is the reduction in depressive symptoms among caregivers, measured using the Patient Health Questionnaire-9 (PHQ-9).^46^ Depression in caregivers is associated with poorer quality of life and increased burden; hence, a reduction in PHQ-9 scores reflects improved caregiver well-being and resilience. The PHQ-9 scores each of the 9 DSM-IV criteria for depression as ‘0’ (not at all) to ‘3’ (nearly every day). A PHQ-9 score of 10 or more has a sensitivity of 88% and specificity of 88% for major depression. Scores of 5, 10, 15 and 20 represent mild, moderate, moderately severe and severe depression. The primary endpoint is the change in PHQ-9 scores from baseline to 8 weeks post-intervention, with an additional follow-up at 6 months (see table 1).

**Table 1:** SPIRIT participant timeline with time schedule of enrolment, intervention implementation and assessment time points

	Study period				
	**Enrolment**	**Allocation**	**Post-allocation**		**Close-out**
Timepoint**	*-t_1_*	**0**	*8 weeks*	*6 months*	*t_x_*
**Enrolment**					
** Eligibility screen**	X				
** Informed consent**	X				
** Allocation**		X			
**Interventions**					
* Better Living with Non-memory-led Dementia*		↔	↔		
* Waitlist control*					
**Assessments**					
* Demographics*	X				
* Clinical outcome measures* ** PHQ-9** ** PSS** ** GAD-7** ** CSES-8** ** EQ-5D-5L** ** ICECAP-A** ** QCPR** ** Resource Use Measure**	X		X	X	
** System Usability Scale (SUS**)			X (intervention group only)		

CSES-8, Caregiver Self-Efficacy Scale; EQ-5D-5L, EuroQol 5-Dimensions, 5-Level ; GAD-7, Generalised Anxiety Disorder-7; ICECAP-A, ICEpop Capability Measure for Adults; PHQ-9, Patient Health Questionnaire-9; PSS, Perceived Stress Scale; QCPR, Quality of Carer–Patient Relationship Scale; SPIRIT, Standard Protocol Items: Recommendations for Interventional Trials.

Secondary outcome measures will be collected at baseline, ~8 weeks (post-intervention, trial endpoint) and 6 months post-baseline (follow-up) (see table 1):

Perceived Stress Scale.^47^ This is the most widely used tool for assessing stress perception. This 14-item tool measures the degree to which situations in one’s life are appraised as stressful. Scores are calculated by reversing responses for four positively stated items (4, 5, 7 and 8) and summing all items. A score of 0–13 indicates low perceived stress, 14–26 moderate and 27–40 high.
Generalised Anxiety Disorder-7 (GAD-7). GAD^48^ is a widely used 7-item scale assessing anxiety symptoms over the past 2 weeks. Endorsed by National Health Sservice (NHS) England’s Improving Access to Psychological Therapies programme as the gold standard measure,^49^ it uses a 0–3 scale (0=not at all, 3=nearly every day). A score of 15 or above indicates severe anxiety.
Caregiver Self-Efficacy Scale (CSES-8).^50^ CSES-8 is an 8-item self-administered measure assessing caregiver confidence in managing caregiving tasks. It uses a 5-point scale (1=strongly disagree, 5=strongly agree). The scale demonstrates high internal consistency (0.89–0.88) and good test-retest reliability (0.73).
EuroQol 5-Dimensions, 5-Level (EQ-5D-5L).^51^ The EQ-5D-5L assesses health-related quality of life across five dimensions**:** mobility, self-care, usual activities, pain/discomfort and anxiety/depression. Each dimension is rated on a 5-level scale from no problems to extreme problems.
ICEpop Capability Measure for Adults (ICECAP-A).^52^ A measure of adult individuals’ freedom to function in five key areas of life, rated on a 4-level scale. It correlates moderately to strongly with the EQ-5D-5L**,** strongly with self-efficacy, and has adequate test-retest reliability (0.79).
Quality of Carer–Patient Relationship Scale (QCPR).^53^ The QCPR is a 14-item measure assessing relationship quality between caregivers and care recipients. It evaluates warmth, conflict and criticism within the caregiving relationship. It has good internal consistency and concurrent validity, and it has been used in research on online interventions for dementia caregivers.

#### Other outcomes

Health and social care resource use, including primary care, outpatient visits, medications and impacts on productivity and daily activities, will be recorded using a participant-reported resource use measure developed as part of this work. An internal pilot (first 10–15 participants) will evaluate its feasibility within the first 2–3 months, with adjustments made if necessary.^54^,^56^ The recommended SPIRIT schedule for participant enrolment, administration of the intervention and assessment time points is shown in table 1.

### Criteria for discontinuation

Participants may discontinue their participation in the study at any time. The data collected to the point of withdrawal will be retained in the analysis set and this is set out explicitly in the participant information document.

### Workstream 2 (WS2): process evaluation

#### Process evaluation

The process evaluation will assess the perceived costs and benefits of the intervention, and mechanisms of change along with participant engagement at ~8 weeks and 6 months post-baseline. It will identify barriers and facilitators affecting engagement, access and implementation. It will be conducted alongside the trial to understand how the BELIDE programme is delivered and experienced by participants and will help interpret trial outcomes and provide insights for potential scalability and adaptation of the intervention. Data sources will include:

- Qualitative interviews with participants (n=30–45) and facilitators to explore their experiences, challenges and perceived benefits of the programme.
- Usage analytics (collected automatically and anonymously by the BELIDE programme) to track engagement with online modules, frequency of access and module completion rates.
- System Usability Scale^57^ used to evaluate the usability of the website (how effectively, efficiently and satisfactorily users can interact with BELIDE’s web platform).
- Facilitator reflections to assess adherence to intervention protocols and barriers to delivery.

### Workstream 3 (WS3): health economics evaluation

The primary analysis will be a cost-utility analysis. The net monetary benefit will be used to summarise QALY (quality-adjusted life year) benefits against willingness to pay thresholds.^58^ A secondary cost-effectiveness analysis will evaluate the incremental costs of achieving a clinically significant improvement in caregiver depressive symptoms (PHQ-9) and will be relevant to healthcare professionals, healthcare decision makers and service users. The base case analysis will be conducted from a societal perspective including impact on ability to work and carry out activities of daily living; a further analysis with a UK NHS and personal social services perspective will be conducted to allow comparison with other published economic evaluations.

A health economic analysis plan will be written following good practice for reporting of the economic evaluation conforming to the Consolidated Health Economic Evaluation Reporting Standards.^59^ Unit costs will be applied to the resource use, as measured in the participant-reported resource use measure, using published unit costs for the cost year for the evaluation including NHS reference costs,^54^ the Personal Social Services Research Unit cost database^55^ and the British National Formulary.^56^ The costs associated with developing and delivering BELIDE, including staff time, materials and equipment, will be collected through structured interviews with the trial team, finance staff and clinical sites as needed.

#### Participant timeline

**Figure 3:**
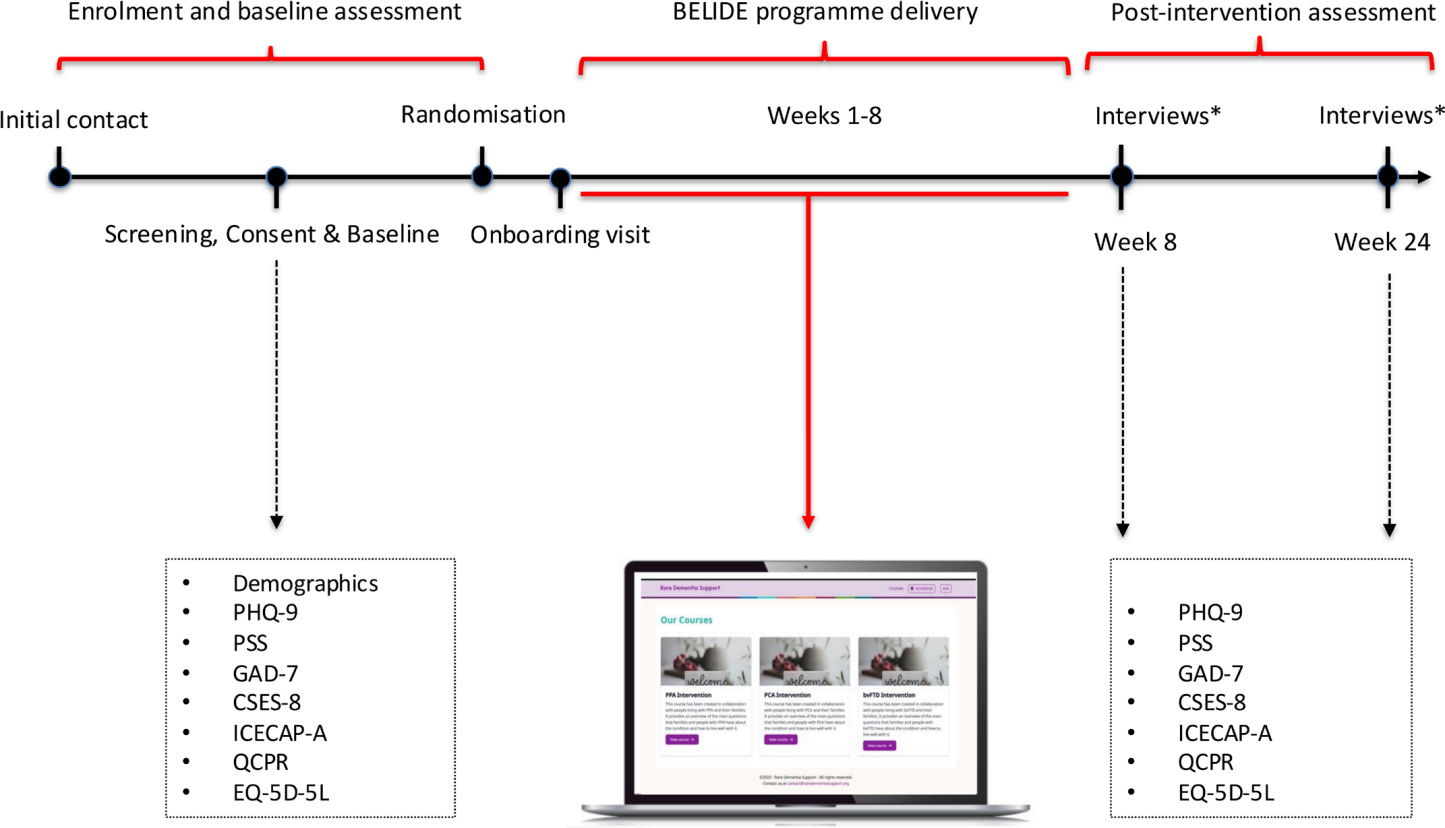
BELIDE’s trial participant timeline. CSES-8, Caregiver Self-Efficacy Scale; EQ-5D-5L, EuroQol 5-Dimensions, 5-Level; GAD-7, Generalised Anxiety Disorder-7; ICECAP-A, ICEpop Capability Measure for Adults; PHQ-9, Patient Health Questionnaire-9; PSS, Perceived Stress Scale; QCPR, Quality of Carer–Patient Relationship Scale.

See figure 3 for a participant timeline showing enrolment, intervention and assessment timepoints.

#### Sample size justification

A total of 238 participants (119 per group) is required to detect a standardised effect size of 0.3 on the primary outcome measure (PHQ-9) at the 8-week follow-up, with 90% statistical power and a significance level of 5%. This sample size allows for a 20% attrition rate, based on an analysis of covariance (ANCOVA) with a coefficient of determination (R^2^) of 0.60 between the primary outcome measure and covariates.

#### Recruitment

The study population consists of supporters, relatives and caregivers of people with dementia who are members of RDS and have opted into its membership database, implying that they accept to be contacted about research opportunities. These individuals may be contacted via email about research opportunities, with clear communication that participation is voluntary and does not affect their RDS membership or care. Participation status will be recorded in members’ internal profiles, accessible only to researchers in this study and the RDS service team. RDS receives 60–100 new sign-ups per month (> 7000 members). Those likely to meet inclusion criteria based on knowledge at sign-up will be invited to participate in the clinical trial. Additionally, we may also approach individuals affiliated with UCL, Bangor, Swansea or King’s College London, including support network members and previous research participants who have consented to be contacted. To enhance accessibility, study advertisements and survey links may be shared on the RDS website and affiliated online platforms, such as social media and collaborator websites.

#### Randomisation

Randomisation will be performed via a secure online platform hosted by the North Wales Organisation for Randomised Trials in Health & Social Care (NWORTH) at Bangor University. Once participants have provided consent and completed baseline measures, they will be entered into the randomisation system. A dynamic adaptive randomisation algorithm will be used to maintain a 1:1 allocation ratio, balanced within stratification variables.^60^ Stratification will be based on diagnostic group (ie, bvFTD, PPA, PCA) and gender identity (ie, male, female, other, prefer not to say).

#### Blinding

Participants in this trial cannot be blinded, given its nature. Individuals who will be analysing data or overseeing the trial including health economists, co-investigators and trial statisticians will remain blind until the blinded analyses detailed in the Statistical Analysis Plan (SAP) have been conducted and reported to the trial team. However, the BELIDE facilitator and investigators leading the process analysis will be unblinded prior to the blinded analyses being conducted. Unblinding will be performed following procedures outlined in NWORTH Standard Opearation Procedure (SOPs).

### Data collection and analysis methods

#### Data collection

Baseline, intervention and follow-up data will be collected using the UCL Qualtrics platform. Participants will complete self-reported questionnaires at baseline, 8 weeks and 6 months post-randomisation. We selected an 8-week primary endpoint to match the intervention duration and assess immediate effects, and a 6-month follow-up to evaluate sustained benefits in line with National Institute for Care and Health Excellence (NICE) guidance. This timeframe is consistent with similar caregiver and digital intervention trials, balancing clinical relevance with retention and data quality. Resource use data will also be collected to evaluate cost-effectiveness. All measures have established reliability and validity. To ensure data quality, automated checks will identify incomplete responses, and research staff will monitor data integrity. Data collection forms are available upon request. Participants who discontinue or deviate from the intervention will still be asked to complete follow-up assessments. Key outcome data will be collected even if full participation in the intervention is not maintained.

The initial onboarding session, subsequent check-in sessions and qualitative interviews will be conducted over an internet-based service (eg, Zoom). BELIDE will be a WordPress website hosted by https://www.cloudnext.uk/ located on UK-based servers and background programme web usage (eg, analytics dashboard embedded in WordPress platform) will also be collected.

#### Data management

Data entry, coding and storage will adhere to UCL data security policies. All data will be securely stored on password-protected servers with restricted access for authorised research staff. Automated range checks will identify inconsistencies, and missing data will be handled using multiple imputation if necessary.

#### Statistical analysis

Primary and secondary outcomes will be analysed on an intention-to-treat (ITT) basis, using linear mixed models, adjusting for baseline values and stratification factors (ie, diagnostic type and gender). The primary outcome, PHQ-9 scores, will be compared between groups at 8 weeks and 6 months post-randomisation. Secondary outcomes, including stress, anxiety, self-efficacy and quality of life, will be analysed in a similar fashion. All estimates of effect will be presented together with 95% CI. A sensitivity analysis will be conducted to assess the impact of the number of times the intervention is accessed. A SAP will be written and signed off before completion of data collection. Missing data will be addressed using multiple imputation techniques where appropriate.

#### Data monitoring

The BELIDE trial is overseen by an independent Data Monitoring Committee (DMC), which monitors trial data and ethics, providing recommendations to the Trial Steering Committee (TSC). The Data Monitoring Committee(DMEC) operates independently from the sponsor, University College London, and further details on its charter are available in governance documents. The DMC will review findings and advise the TSC, which will make the final decision on trial continuation or termination. Trial auditing includes centralised monitoring, internal audits by NWORTH Clinical Trials Unit and biweekly research team meetings. Data are stored securely under General Data Protection Regulation (GDPR) compliance, with access limited to authorised personnel.

##### Adverse events, including serious adverse events

Adverse events will be reported in accordance with UCL Research Ethics Committee (REC) guidelines. Events will be logged, and serious cases will be reported immediately. The main ethical concern in this trial relates to safeguarding issues, particularly around self-harm. A structured safeguarding protocol for suicidality, approved by UCL REC, has been established to manage potential distress participants may experience when reflecting on their role as caregivers of a person with rare dementia. The research will be supervised by AS-G and JS, both qualified clinical psychologists, ensuring compliance with this protocol. In summary, the protocol includes the following: (1) automatic flagging, where Qualtrics will generate an alert for any positive response indicating suicidal ideation; (2) follow-up support, where a research team member will offer a follow-up call within 72 hours; and (3) escalation if needed, this is, if a safeguarding concern is confirmed, the participant will be referred to RDS, where the RDS safeguarding protocol will be applied.

### Patient and public involvement

The BELIDE educational programme was developed following the MRC guidance for development of complex interventions^61^ and its structure and content along with the layout of the digital interface were co-produced with a group of people with lived experience of dementia (see Suárez-González *et al*^37^ for details). In the current trial, Patient and Public Involvement (PPI) will be embedded throughout the duration of the study. PPI co-leads (NZ and VM) will recruit a PPI group among lived experience members of RDS and will support their involvement. All public-facing documents have been developed following templates established with input from RDS members to ensure they are user-friendly and suitable for all levels of literacy skills.

PPI members will contribute to monitoring trial processes (eg, engagement issues), developing and reviewing the topic guide for the process analysis, interpreting research results, producing a plain English summary and planning the dissemination of the study findings. If needed, the trial team will develop and deliver short and simple research methods sessions (eg, ‘what is a randomised controlled trial?’) to help PPI participants understand this specific research process.

## Ethics and dissemination

### Ethical approval and protocol amendments

The study has been approved by the UCL REC (Reference: 8545/007). Any substantial amendments to the protocol will be submitted to the REC for approval before implementation. Changes affecting eligibility criteria, outcomes or data collection will be communicated to investigators, regulatory bodies and trial registries.

### Informed consent

Participants will provide online consent via Qualtrics before screening. If eligible, they will complete a full consent form before proceeding with the study (a copy of the participant consent form is provided as online supplemental file 3). Additional consent will be obtained for post-intervention interviews (a copy of the participant consent form is provided as online supplemental file 4). Participants can withdraw at any time without consequences.

### Confidentiality and data security

All data will be handled per the GDPR (2018). Identifiable information will be stored on UCL’s Data Safe Haven, with access restricted to authorised personnel. Audiovisual data will be pseudonymised, and participant identifiers will be used for secure data management.

### Declaration of interests

The investigators have declared no financial or competing interests related to the study.

### Access to data

The final de-identified dataset will be available through a NIHR-approved data repository 3 months after the grant end date. The data will be stored at the Dementia Research Centre, UCL. Long-term data archiving will be managed in line with UCL’s records office policy.

### Dissemination plan

Trial results will be communicated through peer-reviewed publications, lay summaries, conference presentations, public engagement events and open-access platforms. Findings will be shared with participants through newsletters and stakeholder meetings.

This summary ensures compliance with ethical standards, data protection and transparent research dissemination. Let me know if you need modifications.

## Supplementary material

10.1136/bmjopen-2025-102518 online supplemental file 1

10.1136/bmjopen-2025-102518 online supplemental file 2

10.1136/bmjopen-2025-102518 online supplemental file 3

10.1136/bmjopen-2025-102518 online supplemental file 4

## Data Availability

Data sharing not applicable as no datasets generated and/or analysed for this study.
