# Supplementary material for: Better Living with Non-memory-led Dementia: study protocol for a randomised controlled trial of a web-based caregiver educational programme (BELIDE trial)
Source: BMJ Open. 2025 Sep 5;15(9):e102518. doi: 10.1136/bmjopen-2025-102518 (PMC12414229; doi:10.1136/bmjopen-2025-102518)
Supplement: online supplemental file 2 [file bmjopen-15-9-s002.docx]

**TIDieR (Template for Intervention Description and Replication) Checklist for Better Living with Non-memory Led Dementia (BELIDE) educational programme for supporters of people living with non-memory led dementia.**

| Item Number | Item |
| --- | --- |
| 1. | **Brief name**  Better Living with Non-memory Led Dementia (BELIDE) |
| 2. | **Why**  The BELIDE web-based educational programme aims to improve caregiver well-being and self-efficacy so they can deliver better quality and informed support to their relatives or friends with posterior cortical atrophy (PCA), primary progressive aphasia (PPA) and behavioural variant frontotemporal dementia (bvFTD). BELIDE builds on previous research highlighting the lack of tailored, centralised, accessible and phenotype-tailored resources for caregivers of individuals with these rare dementias. The programme was co-developed with twenty-one people with lived experience (families and people with rare dementias). The co-production process is described in Suárez-González et al., 2023)(1).  The rational is that by equipping caregivers with specialist knowledge, skills and coping strategies, they will feel more confident and effective in their role, reducing stress and depressive symptoms, while enhancing quality of life and better relationships with the person with dementia. This intervention is theory-driven, informed by theories of self-efficacy(2), behaviour change(3), copying theory(4) and social learning(5) that eventually structured the logic model used in the design and development of the programme (see Suárez-González et al., 2023)(1).  The programme offers a blend of self-learning (participant reading through the course material) and human interaction (videocalls with the facilitator). It provides opportunities for reflection, reinterpretation of course material and skill building, incorporating real-life tasks to reinforce engagement and motivation.  By offering a structured yet flexible online format BELIDE aims to provide accessible, evidence-based, phenotype-specific guidance. |
| 3. | **What (Materials used)**  The programme consists of six structured online learning modules, which caregivers can complete at their own pace over an 8-week period. The six modules are:   1. Welcome 2. What is (PCA, PPA, bvFTD, depending on phenotype) 3. Positive Support 4. Living Well 5. Who Can Help 6. Peer Support   The modules sit on the website but can be downloaded in pdf format and printed. See appendix at the end of this table for a detailed description of the content of each module. |
| 4. | **What (Procedures)**  Participants undergo a one-hour virtual onboarding session with a trained facilitator before starting the BELIDE programme. The onboarding session consist of three parts:   1. Interview 2. Introduction to the course (screen sharing) and demonstration of how to use it 3. Personal credentials are sent to the participant, who is asked to log into the course from their own computer while still on the call.   They will complete the six self-paced learning modules indicated above. At the end of module 2,3 and 4, participants are encouraged to complete a real-life practical task (e.g., explaining the disease in lay terms to a friend).  There is a videocall follow up one week after onboarding where participants can ask questions and share their first impressions about the course alongside any strategies they may have used already or are thinking of using. There is one more exchange with the facilitator over email. |
| 5. | **Who provided**  BELIDE is delivered by trained facilitators with a diversity of backgrounds and experience with non-memory-led dementias. It can be delivered by psychologists, assistants and rare dementia support workers. |
| 6. | **How**  BELIDE is delivered completely online, via a web-based platform. Interactions with the facilitator take place via Zoom. The resource is a digital self-paced learning with optional pdf downloads. |
| 7. | **Where**  Sessions are delivered at home or in an outpatient setting, depending on the location of speech and language therapy provision for PPA in a given NHS service. |
| 8. | **When and how much**  BELIDE runs over 8 weeks where participants engage in:   - One hour virtual onboarding session - Six online learning modules (self-paced) - Up to two additional virtual check-in sessions - Participant retain six-month access to content |
| 9. | **Tailoring (Adaptation for Individual Needs)**   - BELIDE is self-paced, allowing caregivers to engage based on their availability. - Modules do not require sequential completion (e.g., participants do not need to complete module one to unlock module two). - Participants are encouraged to skip materials that are not relevant for them and focus on those that are. - Participants can revisit materials at any time. - Participants can ask facilitators questions or request clarifications |
| 10. | **Modifications (Changes during the trial)**  No planned modifications at this stage, but process evaluation will explore barriers, facilitators, and potential refinements. |
| 11. | **How well**  No assessment of fidelity planned |

# Appendix. Detailed Manuals Content Table

| Section | Better Living with PPA | Better Living with PCA | Better Living with bvFTD |
| --- | --- | --- | --- |
| Welcome | General introduction | | |
| What is the condition? | 1. What is PPA? 2. How is PPA diagnosed? 3. How is PPA different from Alzheimer's disease? 4. Is PPA a common condition? 5. What causes PPA? 6. Types of PPA 7. Symptoms 8. Not everything is language 9. Carry an 'I HAVE PPA' card 10. Put your knowledge into practice | 1. How is PCA diagnosed? 2. How is PCA different from Alzheimer's disease? 3. Is PCA a common condition? 4. What causes PCA? 5. Symptoms 6. Why these symptoms and not others? 7. Carry an 'I HAVE PCA' card 8. Put your knowledge into practice | 1. What is bvFTD? 2. How is bvFTD diagnosed? 3. How is bvFTD different from Alzheimer's disease? 4. Is bvFTD a common condition? 5. What causes bvFTD? 6. Symptoms 7. Why these symptoms and no others? 8. Carry an 'I HAVE bvFTD' card and the sunflower lanyard 9. Put your knowledge into practice |
| Positive Support | 1. Positive Support  2. Help someone with PPA to improve their communication skills  3. How to speak to someone with PPA to help with their understanding  4. How to respond supportively when someone with PPA shows problems communicating  5. Non-verbal activities  6. Learn more  7. Put your knowledge into practice | 1. Positive Support  2. Visual difficulties  3. Problems with space and body positioning  4. Reduced hand dexterity and dressing difficulties  5. Learn more  6. Put your knowledge into practice | 1. Positive support  2. Irritability and angry reactions  3. Socially inappropriate behaviour  4. Loss of interest in activities  5. Repetitive thinking behaviour  6. Thinking rigidity  7. Excessive eating or drinking  8. Learn more  9. Put your knowledge into practice |
| Living Well | 1. Living well 2. Finding companions and investing in support 3. Strategies for stress management 4. Meaningful and enjoyable activities 5. Responding to changes in behaviour 6. Put your knowledge into practice | | |
| Who Can Help? | Support services and professional help | | |
| Peer Support | Connecting with others with PPA | Connecting with others with PCA | Connecting with others with bvFTD |
