# Supplementary material for: Better Living with Non-memory-led Dementia: study protocol for a randomised controlled trial of a web-based caregiver educational programme (BELIDE trial)
Source: BMJ Open. 2025 Sep 5;15(9):e102518. doi: 10.1136/bmjopen-2025-102518 (PMC12414229; doi:10.1136/bmjopen-2025-102518)
Supplement: online supplemental file 3 [file bmjopen-15-9-s003.pdf]

Better Living with Non-memory Led Dementia – Randomised Clinical trial

Full Consent Form

Full title of project: Living Better With Rare Dementias: Testing blended person/digital intervention for carers of rare dementia to improve psychological outcomes
Project number: 8545.007
Name of lead investigator: Prof. Joshua Stott

Trial Lead: Dr. Aida Suarez Gonzalez

Please tick each of these statements below to indicate your consent to each point:

1. I confirm that I have read and understood the information sheet dated XXXX for this clinical trial, had the opportunity to ask questions and have had these answered satisfactorily.
2. I understand that my participation is voluntary and that I am free to withdraw at any time, without giving any reason. I understand that if I withdraw this will not affect my health care or my legal rights in any way.
3. I understand that if I withdraw from the study the research team may continue to use the information that I previously provided up to that point.
4. I understand that the information collected about me may be used to support other research in the future and may be shared (without any details that could personally identify me) with other researchers.
5. I understand that I will not be identifiable in any data published in relation to this project.
6. I understand this study requires my involvement for six months and that I will be contacted by the research team over that period in relation to this research after today's date.
7. I understand that if the researchers hear or observe anything that causes serious concern about my health, safety, or well-being, or that of another person close to me, they have a duty to inform the lead investigator and any relevant authorities.
8. I understand that I will be offered a £15 voucher as a token of appreciation for the time given to participation in the study.
9. I agree that a copy of my data (which does not contain any personal information about me) can be deposited and securely stored in a data archive.
10. I agree to take part in the above study.

Name of Participant_______________________________________

Date________________
