# Supplementary material for: Better Living with Non-memory-led Dementia: study protocol for a randomised controlled trial of a web-based caregiver educational programme (BELIDE trial)
Source: BMJ Open. 2025 Sep 5;15(9):e102518. doi: 10.1136/bmjopen-2025-102518 (PMC12414229; doi:10.1136/bmjopen-2025-102518)
Supplement: online supplemental file 4 [file bmjopen-15-9-s004.pdf]

Discussing my experiences of using Better Living with Non-memory Led Dementia

Consent Form

Full title of project: Living Better With Rare Dementias: Testing blended person/digital intervention for carers of rare dementia to improve psychological outcomes
Project number: 8545.007
Name of lead investigator: Prof. Joshua Stott

Trial Lead: Dr. Aida Suarez Gonzalez

[There are threeways that the following questions can be answered. The first is where participants are asked all the below questions on a recorded videoconference call and their verbal consent to this is recorded. The second is where participants send a completed consent form via email that they agree to all conditions below ,and the third is to send a completed consent form via freepost. All questions regarding the study can be directed to our trial mailbox: betterliving@ucl.ac.uk where a member of the research team can respond.]

Participant identification number:_______________

1. I confirm that I have read and understood the information sheet dated XXXX for this clinical trial, had the opportunity to ask questions and have had these answered satisfactorily.
2. I understand that my participation in this interview is voluntary and that I am free to withdraw at any time, without giving any reason. I understand that if I withdraw this will not affect my health care or my legal rights in any way.
3. I understand that if I refuse to take part in this post-intervention interviews or I withdraw from the study the research team may continue to use the information that I previously provided up to that point.
4. I understand that the information collected about me may be used to support other research in the future and may be shared (without any details which could personally identify me) with other researchers.
5. I understand that I will not be identifiable in any data published in relation to this project.
6. I understand this study requires my involvement over one interview session.
7. I understand that if the researchers hear or observe anything that causes serious concern about my health, safety, or well-being, or that of another person close to me, they have a duty to inform the lead investigator and any relevant authorities.
8. I understand that my data may be accessed as part of audit or quality control processes by regulatory bodies where necessary to carry out this process.
9. I agree that a copy of my data that does not contain any personal identifying information about me can be deposited and securely stored in a data archive.
10. I agree to take part in this additional research activity relating to the **Better Living with Non-memory Led Dementia** study.

Name of Participant____-

Date__________________

Name of person taking consent____--
Date__________________
