# Supplementary material for: Better Living with Non-memory-led Dementia: study protocol for a randomised controlled trial of a web-based caregiver educational programme (BELIDE trial)
Source: BMJ Open. 2025 Sep 5;15(9):e102518. doi: 10.1136/bmjopen-2025-102518 (PMC12414229; doi:10.1136/bmjopen-2025-102518)
Supplement: Supplementary file 5 [file bmjopen-2025-102518.reviewer_comments.pdf]

PEER REVIEW HISTORY

BMJ Open publishes all reviews undertaken for accepted manuscripts. Reviewers are asked to complete a checklist review form and are provided with free text boxes to elaborate on their assessment. These free text comments are reproduced below.

ARTICLE DETAILS

Title (Provisional)

Better Living with Non-Memory-Led Dementia: Study Protocol for a Randomised Controlled Trial of a Web-Based Caregiver Educational Programme (BELIDE Trial)

Authors

Suarez-Gonzalez, Aida; Brotherhood, Emilie V.; John, Amber; Hayes, Oliver; Rossi-Harries, Sam; Zimmermann, Nikki; Mansfield, Valerie; Brand, Andrew; Hoare, Zoe; Fitzsimmons, Deborah; Cullen, Katherine; Crutch, Sebastian; Stott, Joshua

---

VERSION 1 - REVIEW

**Reviewer**	**1**
**Name**	**Ali, Siti**
**Affiliation**	**Universiti Sains Malaysia, Neuroscience**
**Date**	**01-Jun-2025**
**COI**	**None**

Strengths of the review

This article comes at the very need of time where topic is highly relevant and novel, addressing a critical service gap in dementia caregiving, specifically for non-memory-led dementias (PPA, PCA, bvFTD)

Abstract:

- The abstract is slightly too long and dense for quick understanding.
- It lacks a final sentence discussing the potential impact or novelty of the trial outcomes
- Line 11-The programme name BELIDE should be consistently formatted (e.g: "Better Living with
  Non-Memory-Led Dementia (BELIDE)" at first mention and not repeated unnecessarily.

Introduction:

- The introduction would benefit from a more comprehensive epidemiological context regarding the prevalence and diagnosis challenges of non-memory-led dementias, especially outside the UK.
- While the co-design approach is briefly mentioned, further detail on pilot testing outcomes could enhance the narrative flow.

- The research questions are clear but may be strengthened by explicitly linking each question back to the gaps identified earlier in the introduction.
- The references used are highly relevant and predominantly up to date. However, a few older references (ex: Cohen et al., 1983; Bandura, 1999) are theoretically justifiable, but could be supplemented with more recent empirical validation of those measures in online or dementia- caregiver contexts.
- It would be useful to include recent emerging literature on digital interventions in dementia caregiving from outside Europe for greater global relevance.

Methods:

- Clear description of intervention content (TIDieR), control condition, and blinding protocol enhances methodological transparency.
- Inclusion/exclusion criteria are appropriate and support ecological validity.
- Although the outcome measures are validated, the primary endpoint is only at 8 weeks. While a 6-month follow-up is included, further justification could be provided for why longer-term effects are not explored, especially for web-based interventions.
- The protocol would benefit from more clarity on handling potential contamination. For example, if control participants access public resources overlapping with BELIDE content).

Discussion

- The manuscript would be enhanced by briefly discussing how BELIDE compares innovation or scalability to existing interventions like RHAPSODY or Partner in Balance beyond stating that they are not fully powered RCTs.
- There is limited reflection on potential limitations of generalisability. For example, recruiting from a support organisation may bias toward more proactive caregivers).

**Reviewer**	**2**
**Name**	**Dsouza, Sebestina**
**Affiliation**	**Manipal Academy of Higher Education, Occupational Therapy**
**Date**	**18-Jun-2025**
**COI**	**None**

This is a well conceptualized, planned and written RCT protocol. Just two small issues. As required by BMJ, the dates of the study (planned/ongoing) need to be included in the study.

There are few typo errors

1. pg 9, line 45...citation

2. there are two Figure 2 on page 9 (BELIDE) and page 10 (SPIRIT). Please check and correct all figures and tables as appropriate.

Best wishes with the study

VERSION 1 - AUTHOR RESPONSE

Reviewer 1 (Dr. Siti Ali): Abstract: • The abstract is slightly too long and dense for quick understanding. • It lacks a final sentence discussing the potential impact or novelty of the trial outcomes • Line 11-The programme name BELIDE should be consistently formatted (e.g: “Better Living with Non-Memory-Led Dementia (BELIDE)” at first mention and not repeated unnecessarily.

#Response: Thank you for these very helpful comments. We have substantially shortened the Introduction section of the abstract, which we think makes it more clear and accessible. We have added a final sentence discussing the potential impact of the trial outcome: “If effective, this programme could improve access to caregiver support for non-memory-led dementias by providing scalable, tailored education.” We have consistently used BELIDE across the manuscript and removed repetitions to the fully spelled named of the programme across the manuscript.

Reviewer 1 (Dr. Siti Ali): The introduction would benefit from a more comprehensive epidemiological context regarding the prevalence and diagnosis challenges of non-memory-led dementias, especially outside the UK.

#Response: We appreciate the reviewer’s suggestion to expand on the epidemiological context of non-memory-led dementias. While we agree that global prevalence and diagnostic challenges are important issues, we feel that a detailed exploration of these aspects would go beyond the intended scope of this protocol paper, which is specifically focused on the evaluation of an online intervention. Nonetheless, we have ensured the introduction provides sufficient context to justify the study aims and target population.

Reviewer 1 (Dr. Siti Ali): While the co-design approach is briefly mentioned, further detail on pilot testing outcomes could enhance the narrative flow.

#Response: Thank you for this remark. We have added the following to the introduction: “A previous pilot study demonstrated successful recruitment, high completion rates of outcome measures, and good acceptability of the BELIDE programme, supporting its use in a fully powered trial”

Reviewer 1 (Dr. Siti Ali): The research questions are clear but may be strengthened by explicitly linking each question back to the gaps identified earlier in the introduction.

#Response: We thank the reviewer for this helpful suggestion. While we agree that linking each research question directly to the identified gaps could aid clarity, we felt that doing so within the Objectives and Research Questions section would risk

unnecessary repetition and affect the readability of the text. Instead, we ensured that the research questions are tightly aligned with the rationale developed in the preceding paragraphs, allowing the connection to be clear without restating prior content.

Reviewer 1 (Dr. Siti Ali): The references used are highly relevant and predominantly up to date. However, a few older references (ex: Cohen et al., 1983; Bandura, 1999) are theoretically justifiable, but could be supplemented with more recent empirical validation of those measures in online or dementia-caregiver contexts.

#Response: We appreciate the reviewer's careful reading of our references. The cited works by Cohen et al. (1983) and Bandura (1999) are not used to justify outcome measures per se, but rather to ground the theoretical constructs—perceived stress and self-efficacy—that underpin our study. These are foundational sources that continue to be widely cited and remain central to the understanding of stress and behavioural interventions in caregiving contexts.

Reviewer 1 (Dr. Siti Ali): It would be useful to include recent emerging literature on digital interventions in dementia caregiving from outside Europe for greater global relevance.

#Response: We agree on the importance of global relevance. However, the literature on digital interventions specifically for caregivers of non-memory-led dementias remains limited, and the references cited represent all currently available studies in this area, regardless of region.

Reviewer 1 (Dr. Siti Ali): Clear description of intervention content (TIDieR), control condition, and blinding protocol enhances methodological transparency.

#Response: We thank the reviewer for highlighting the clarity of our methodological reporting.

Reviewer 1 (Dr. Siti Ali): Inclusion/exclusion criteria are appropriate and support ecological validity.

#Response: We thank the reviewer for their positive comment.

Reviewer 1 (Dr. Siti Ali): Although the outcome measures are validated, the primary endpoint is only at 8 weeks. While a 6-month follow-up is included, further justification could be provided for why longer-term effects are not explored, especially for web-based interventions.

#Response: Thank you for this comment. We selected an 8-week primary endpoint,

and a 6-month follow-up based on several considerations. First, the 8-week primary endpoint corresponds to the duration of the intervention, allowing us to assess immediate post-intervention effects. It aligns with the average length of similar psychosocial and educational interventions for caregivers, as shown in the references cited in the manuscript. Second, a 6-month follow-up is included to assess whether benefits are maintained over time, complying with the NICE (National Institute for Health and Care Excellence) guidance for psychosocial interventions in dementia, which recommends evaluating outcomes at a minimum of 6 months to assess meaningful clinical impact. Additionally, 6 months is widely used as a standard timeframe in trials of digital interventions and caregiver programmes. It offers a balance between capturing sustained effects and minimising attrition. Longer-term follow-up often suffers from increased drop-out rates and reduced data quality, particularly in web-based trials involving unpaid carers of people with progressive conditions. As a result, we prioritised methodological rigour and retention by focusing on a timeframe that is both realistic and clinically meaningful.

Reviewer 1 (Dr. Siti Ali): The protocol would benefit from more clarity on handling potential contamination. For example, if control participants access public resources overlapping with BELIDE content).

#Response: We acknowledge the potential for contamination through control participants accessing publicly available resources. To minimise this, participants in the control group are only signposted to the Rare Dementia Support (RDS) website, the main publicly available source for this population. The BELIDE programme includes curated, structured, and interactive modules not available through RDS or other public resources. Additionally, we collect information at each time point on any external support or educational materials accessed by participants, which will allow us to explore potential contamination in sensitivity analyses.

Reviewer 1 (Dr. Siti Ali): Discussion • The manuscript would be enhanced by briefly discussing how BELIDE compares innovation or scalability to existing interventions like RHAPSODY or Partner in Balance beyond stating that they are not fully powered RCTs.
#Response: We thank the reviewer for this helpful suggestion. As this is a protocol paper, and consistent with standard practice, the manuscript does not include a full discussion section. However, we acknowledge the value of the reviewer's point and have added a brief comparison at the end of the Web-based educational resources section that reads: "Moreover, Partner in Balance requires ~ 3 hours of facilitator contact per caregiver and prior facilitator training [34], while Rhapsody is fully self-guided[35] Human support may be needed to ensure engagement, as suggested by lower uptake in RHAPSODY and our own feasibility work[37], but its costs can limit sustainability.. Finding the right balance in the amount of facilitator involvement seems key to effective implementation and roll out."

Reviewer 1 (Dr. Siti Ali): There is limited reflection on potential limitations of generalisability. For example, recruiting from a support organisation may bias toward more proactive caregivers).

#Response: We appreciate this valuable observation. As this is a protocol paper, it does not include a formal discussion section, in line with standard practice. However, we fully agree that the potential impact on generalisability is an important consideration. We have therefore added this point to the 'Strengths and limitations' section.

Reviewer 2 (Prof. Sebestina Dsouza): As required by BMJ, the dates of the study (planned/ongoing) need to be included in the study.

#Response: Thank you for this comment. We have added the following to the first paragpah of the Methods and Analysis section:

"The study commenced in January 2024 and is expected to conclude in September 2027, with primary outcome data collection ending in March 2026."

Reviewer 2 (Prof. Sebestina Dsouza): There are few typo errors: 1. pg 9, line 45...citation
2. there are two Figure 2 on page 9 (BELIDE) and page 10 (SPIRIT). Please check and correct all figures and tables as appropriate.

#Response: We thank the reviewer for their careful reading. We have thoroughly checked the manuscript for typographical errors.
